# A case of tuberous sclerosis complex with concomitant primary hyperparathyroidism due to parathyroid adenoma: a case report

**DOI:** 10.1186/s12957-015-0520-y

**Published:** 2015-03-13

**Authors:** Yuji Shinzato, Yasukazu Ikehara

**Affiliations:** Department of Pediatrics, Chubu Tokushukai Hospital, Teruya 3-20-1, Okinawa City, Okinawa Prefecture 904-8585 Japan; Department of Surgery, Chubu Tokushukai Hospital, Teruya 3-20-1, Okinawa City, Okinawa Prefecture 904-8585 Japan

**Keywords:** Tuberous sclerosis complex, Primary hyperparathyroidism, Hypercalcemia, Hypophosphatemia, Parathyroid adenoma, Neuroendocrine tumors

## Abstract

The patient was a 27-year-old woman who was clinically diagnosed with tuberous sclerosis complex (TSC). She developed hypercalcemia and hypophosphatemia at age 23. In a detailed examination at age 26, she was diagnosed with primary hyperparathyroidism due to parathyroid adenoma. After undergoing parathyroidectomy, her hypercalcemia and hypophosphatemia rapidly normalized. Subsequent genetic testing revealed mutations of the *TSC1* gene. TSC with concomitant parathyroid adenoma is extremely rare; only three cases have been reported worldwide. However, each of these cases was diagnosed clinically. Therefore, our case is the first to be diagnosed with genetic testing.

## Background

Tuberous sclerosis complex (TSC) is an autosomal dominant disorder that results from an inactivating mutation in one of two genes, *TSC1* (on chromosome 9q34), which encodes hamartin, or *TSC2* (on chromosome 16p13.3), which encodes tuberin.

Hamartin and tuberin form a complex that regulates cell proliferation and differentiation [1]. In TSC, hamartomas and neoplastic lesions form at various sites throughout the body. Lesions are often observed on the central nervous system, eyes, skin, kidneys, heart, or lungs; however, concomitant parathyroid adenoma is extremely rare and currently unreported in Japan. To date, only three cases have been reported in the entire world [2-4]. However, each of these cases was diagnosed clinically. Indeed, there is no reported case of TSC that was diagnosed with genetic testing. Here, we report our experience with a highly rare case of TSC with concomitant primary hyperparathyroidism due to parathyroid adenoma.

## Case presentation

### Clinical course

The patient was a 27-year-old woman who began having convulsive seizures at age 10. Her seizure types were consistent with the following categories: generalized tonic-clonic convulsion, absence seizure, and epileptic automatism.

She was found to have cortical tubers, subependymal nodules, retinal hamartoma, right ventricular cardiac rhabdomyoma, white leaf-shaped macules on the right thigh, and shagreen patches on the lower back. No intellectual disability was present, and she had no family history of TSC. She was clinically diagnosed with TSC. Although her epilepsy was initially intractable, the attacks eventually decreased and stopped occurring at age 17. A cyst was discovered on the right kidney at age 19, followed by multifocal micronodular pneumocyte hyperplasia and hepatic hemangioma at age 26. However, both were asymptomatic and did not show a tendency toward enlargement; therefore, the patient’s course is currently being observed while she receives only sodium valproate.

**Figure 1:**
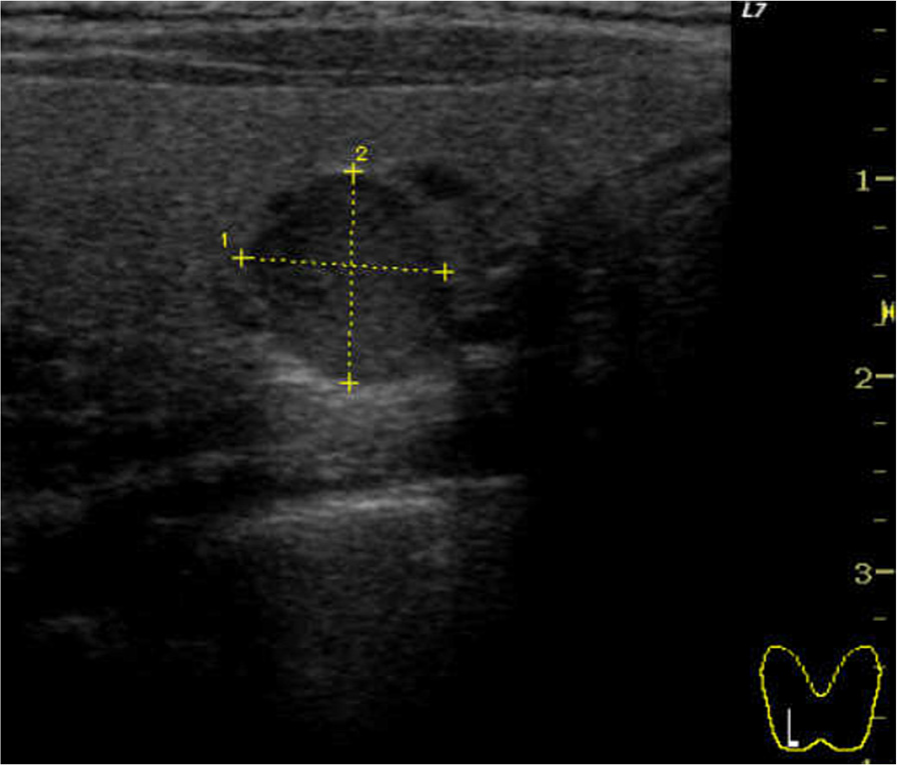
**Cervical ultrasonography.** A mass shadow with a 1-cm diameter at a site corresponding to the right inferior pole of the thyroid gland.

**Figure 2:**
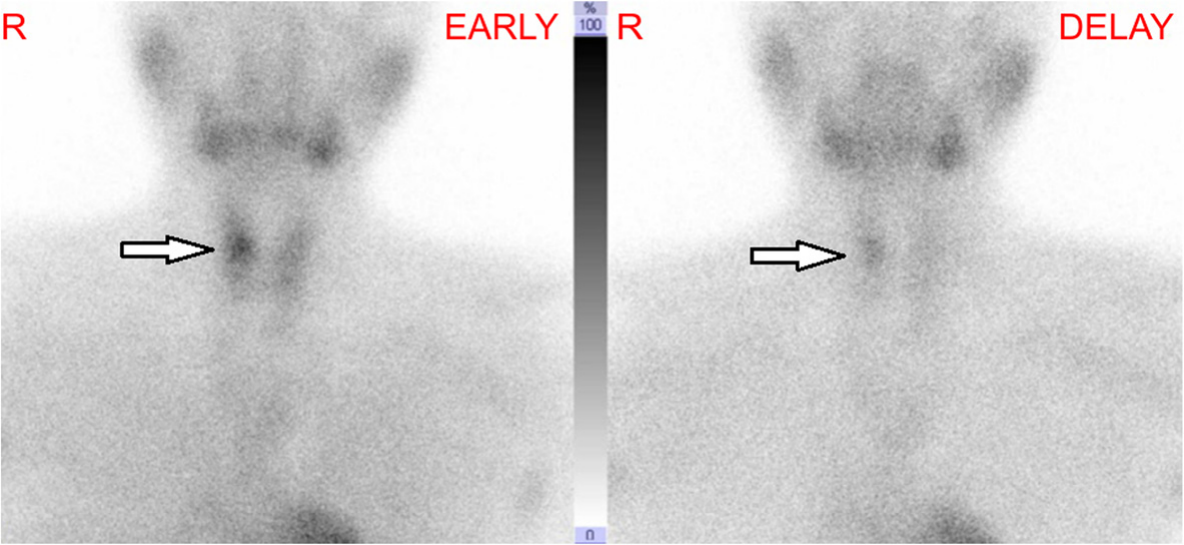
**Technetium-99m methoxy-isobutyl isonitrile (Tc99m MIBI) scintigraphy.** This image shows accumulation at the same site that had been identified through ultrasonography.

**Figure 3:**
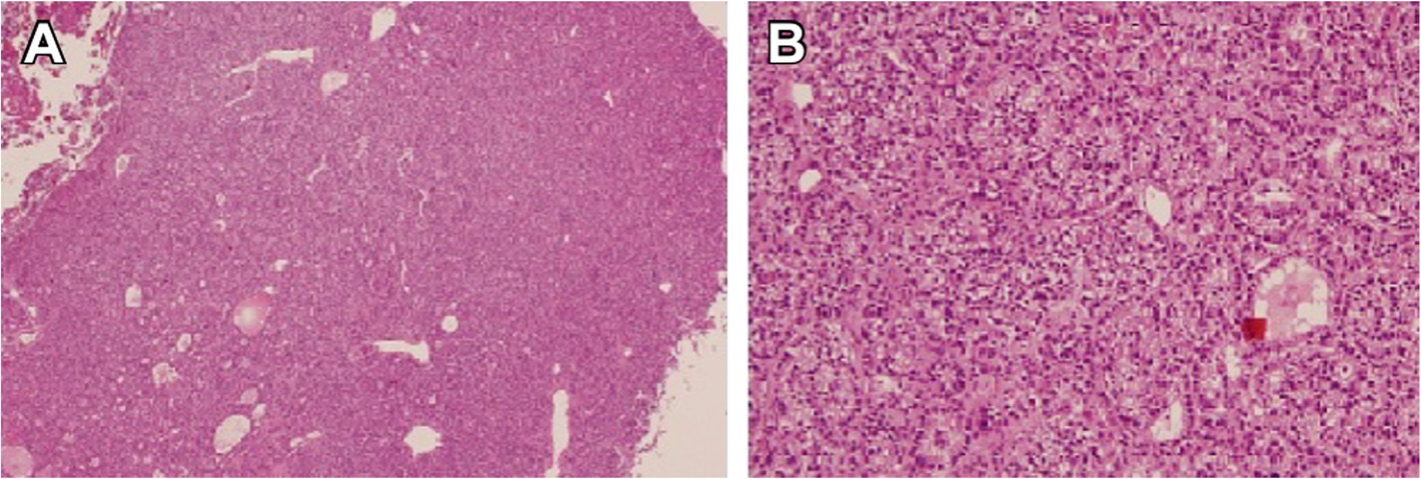
**Parathyroid adenoma resection sample.** The sample weighed 0.3 g. We observed densely hyperplastic chief primary cells without fat cells. No atypia or evidence of infiltration is seen. Although a normal rim cannot be seen, the sample was considered a parathyroid adenoma based on the enlargement of one gland. **A** (Left): ×4; **B** (Right): ×10. Hematoxylin and eosin staining.

Hypercalcemia and hypophosphatemia developed and gradually progressed from age 23, according to regular annual testing (Table 1). She has shown no symptoms of hypercalcemia, such as fracture or renal stone. Bone mineral density (BMD) of the lumbar spine was normal (T-score: −0.7) and renal function was also normal (creatinine: 0.55 mg/dL). Thus, when the patient underwent detailed examination at age 26, she was diagnosed with asymptomatic primary hyperparathyroidism with increased parathyroid hormone activity (intact PTH) (Table 2). The results of tests of thyroid function, gastrin, insulin glucagon, vasoactive intestinal peptide, and prolactin were all normal. Cervical ultrasonography showed a mass shadow with a 1-cm diameter at a site corresponding to the right inferior pole of the thyroid gland. Technetium-99m methoxy-isobutyl isonitrile (Tc99m MIBI) scintigraphy demonstrated accumulation at the same site that had been identified through ultrasonography (Figures 1 and 2). She underwent minimally invasive parathyroidectomy (PTx). The resected parathyroid adenoma weighed 0.3 g, and we observed densely hyperplastic chief primary cells without fat cells. No atypia or evidence of infiltration was seen. Although a normal rim could not be observed, the sample was considered a parathyroid adenoma based on the enlargement of one gland (Figure 3). Following surgery, the hypercalcemia, hypophosphatemia, and the plasma intact PTH level rapidly normalized. These values currently remain at normal levels, 1 year after PTx (Table 3).

**Table 1:** **Annual transitions in serum calcium and phosphorus**

**Age (years)**	**23**	**24**	**25**	**26**
Ca (mg/dL)	10.7	10.9	11.3	11.5
P (mg/dL)	2.8	2.3	2.3	1.9
Albumin (g/dl)	Not performed	Not performed	4.3	4.4

*Ca* calcium, *P* phosphorous.

**Table 2:** **Endocrinological test findings**

**Serum laboratory studies**	**Observed values**	**Normal values**
Intact PTH (pg/mL)	147	10–65
PTHrp (pmol/L)	<1.1	0–1
1-25(OH)_2_D (pg/mL)	55	20–60
25(OH)D(ng/mL)	10.8	9–33.9

*PTH* parathyroid hormone, *PTHrp* parathyroid hormone-related protein-C, *1*-*25(OH)2D* 1,25-dihydroxyvitamin D_3_, *25(OH)D* 25-hydroxyvitamin D.

**Table 3:** **Serum calcium, phosphorus, and PTH level after PTx**

**After PTx**	**1 day**	**2 weeks**	**1 month**	**3 months**	**6 months**	**1 year**
Ca (mg/dL)	9.0	9.3	9.1	9.8	9.7	9.4
P (mg/dL)	2.5	3.3	2.7	2.5	2.5	3.3
Intact PTH (pg/mL)	9	43	42	27	38	39

*Ca* calcium, *P* phosphorus, *PTH* parathyroid hormone, *PTx* parathyroidectomy.

**Figure 4:**
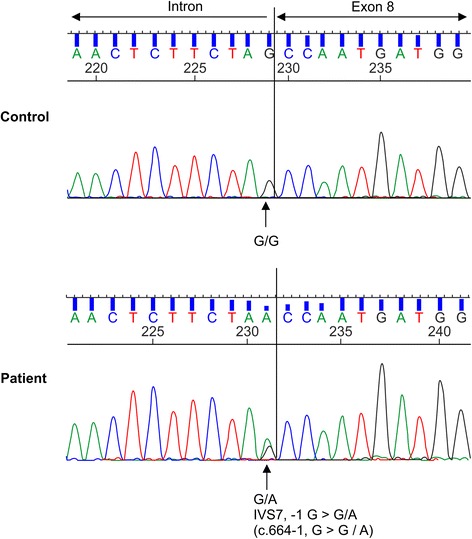
**Gene analysis results.** A novel splicing mutation in the *TSC1* gene. Mutated and normal nucleotides (A and G, respectively) were present in the patient.

Previously, the patient had been clinically diagnosed with TSC. At this point, we decided that a genetic test should also be performed because new complications had been found, such as parathyroid adenoma. In the parathyroid adenoma, the *TSC* gene may have undergone loss of heterozygosity (LOH). A novel splicing mutation was revealed by direct sequencing analysis of the *TSC1* gene extracted from peripheral blood. This gene abnormality occurred at the location of an important consensus sequence of the splicing site, and the probability of a gene splicing abnormality is high. Thus, we surmised that the mutation might be pathogenic (Figure 4). The results of the analysis of the *TSC2* gene were normal. Genetic testing was performed by the Research Center for Bioscience and Technology at Tottori University.

## Discussion

It is thought that the prevalence of TSC does not differ according to region or race [5,6]. Exact data on the prevalence of TSC in Japan are not available, but according to a local investigation, the prevalence is estimated to be at least about 1 in 6,000 persons. Thus, it is supposed that there are about 15,000 patients with TSC in Japan [6].

The incidence of primary hyperparathyroidism is considered to be 1 in 2,500 to 5,000 persons in Japan [7]. Asymptomatic cases, such as our case, are often detected because of the increasingly common use of blood tests, including those of calcium (Ca), phosphorous (P), and intact PTH.

TSC is an autosomal dominant disease that occurs because of abnormalities in the *TSC1* or *TSC2* tumor suppressor genes. Tumorigenesis in TSC is explained by Knudson’s two-hit mutational mechanism [8]. The first hit corresponds to a germline mutation inactivating one of the alleles of either *TSC1* or *TSC2*, and the second hit, called LOH, is a somatic mutation inactivating the second allele [9,10]. In fact, LOH has been confirmed in the development of multiple hamartomas in a variety of organs and tissues in TSC patients [9,11-13].

*TSC1* encodes hamartin and *TSC2* encodes tuberin. *TSC1* and *TSC2* form a complex that regulates mammalian target of rapamycin (mTOR) activity in an inhibitory fashion. mTOR itself regulates cell growth and proliferation [14]. Thus, it is believed that mTOR increases if the hamartin-tuberin complex is functionally impaired, resulting in dysplasia, tumor formation, and angiogenesis due to accelerated cell proliferation, and thereby causing the systemic manifestation of various symptoms [10,15,16]. mTOR inhibitors have recently been found to be effective for subependymal giant cell astrocytoma, renal angiomyolipoma, and pulmonary lymphangioleiomyomatosis, which are severe manifestations of TSC [9,16,17].

Although cases of TSC rarely include concomitant neuroendocrine tumors of the pituitary gland, parathyroid, pancreas, or adrenal gland, such cases have been reported [3,18,19]. Because these cases are so infrequent, they tend to be viewed as coincidental. However, there are confirmed cases of *TSC2* gene LOH with pancreatic neuroendocrine tumors accompanying TSC [18,19]. In addition, researchers investigating non-familial pancreatic neuroendocrine tumors have recently detected mutations of the *TSC2* gene that affect the mTOR pathway in a manner that is similar to the mutations of phosphatase and tensin homolog deleted on chromosome 10 (*PTEN*) and phosphatidylinositol-4,5-bisphosphate 3-kinase, catalytic subunit alpha (*PIK3CA*) [20]. Note that these *TSC2* gene mutations have not been detected as frequently as multiple endocrine neoplasia type 1 (*MEN1*) and death domain-associated protein/alpha thalassemia/mental retardation syndrome X-linked (*DAXX*/*ATRX*) gene mutations [20]. Therefore, neuroendocrine tumors are considered to be possible complications of TSC. In the present case, we attempted to investigate whether LOH had occurred in the resected parathyroid adenoma tissue; however, analysis was not possible due to the poor state of the sample. Although there are no reports of LOH of the *TSC* gene in parathyroid tissue, future cases are expected.

## Conclusions

Our case suggests that it is possible for primary hyperparathyroidism to be complicated with TSC, even though this presentation is extremely rare.

## Consent

Written informed consent was obtained from the patient for publication of this case report and any accompanying images. A copy of the written consent is available for review by the Editor-in-Chief of this journal.

## Abbreviations

1-25(OH)_2_D: 1,25-dihydroxyvitamin D_3_
25(OH)D: 25-hydroxyvitamin D
ATRX: alpha thalassemia/mental retardation syndrome X-linked
BMD: bone mineral density
Ca: calcium
DAXX: death domain-associated protein
LOH: loss of heterozygosity
MEN1: multiple endocrine neoplasia type 1
mTOR: mammalian target of rapamycin
P: phosphorous
PIK3CA: phosphatidylinositol-4,5-bisphosphate 3-kinase, catalytic alpha
PTEN: phosphatase and tensin homolog deleted on chromosome 10
PTH: parathyroid hormone
PTHrp: parathyroid hormone-related protein-C
PTx: parathyroidectomy
Tc99m MIBI: technetium-99m methoxy-isobutyl isonitrile
VIP: vasoactive intestinal peptide
